# Synergy, Additivity, and Antagonism between Cisplatin and Selected Coumarins in Human Melanoma Cells

**DOI:** 10.3390/ijms22020537

**Published:** 2021-01-07

**Authors:** Paula Wróblewska-Łuczka, Aneta Grabarska, Magdalena Florek-Łuszczki, Zbigniew Plewa, Jarogniew J. Łuszczki

**Affiliations:** 1 Department of Pathophysiology, Medical University of Lublin, 20-090 Lublin, Poland; paula.wroblewska-luczka@umlub.pl; 2 Department of Biochemistry and Molecular Biology, Medical University of Lublin, 20-090 Lublin, Poland; aneta.grabarska@umlub.pl; 3 Department of Medical Anthropology, Institute of Rural Health, 20-950 Lublin, Poland; magdalena.florek@wp.pl; 4 Department of General, Oncological, and Minimally Invasive Surgery, 1 Military Clinical Hospital with the Outpatient Clinic in Lublin, 20-400 Lublin, Poland; plewa_z@o2.pl

**Keywords:** melanoma, cisplatin, coumarins, drug interaction

## Abstract

(1) Cisplatin (CDDP) is used in melanoma chemotherapy, but it has many side effects. Hence, the search for natural substances that can reduce the dose of CDDP, and CDDP-related toxicity, is highly desired. Coumarins have many biological properties, including anticancer and antiproliferative effects. (2) An in vitro 3-(4,5-dimethylthiazol-2-yl)-2,5-diphenyl-2H-tetrazolium bromide (MTT) assay on two human melanoma cell lines (FM55P and FM55M2) examined the antitumor properties of CDDP and five naturally occurring coumarins (osthole, xanthotoxin, xanthotoxol, isopimpinellin, and imperatorin). The antiproliferative effects produced by combinations of CDDP with the coumarins were assessed using type I isobolographic analysis. (3) The most potent anticancer properties of coumarins were presented by osthole and xanthotoxol. These compounds were characterized by the lowest median inhibitory concentration (IC_50_) values relative to the FM55P and FM55M2 melanoma cells. Isobolographic analysis showed that for both melanoma cell lines, the combination of CDDP and osthole exerted synergistic and additive interactions, while the combination of CDDP and xanthotoxol exerted additive interactions. Combinations of CDDP with xanthotoxin, isopimpinellin, and imperatorin showed antagonistic and additive interactions in two melanoma cell lines. (4) The combination of CDDP and osthole was characterized by the most desirable synergistic interaction. Isobolographic analysis allows the selection of potential candidates for cancer drugs among natural substances.

## 1. Introduction

Cancer, with over 11 million deaths annually, is the leading cause of death in economically developed countries and the second leading cause of death in developing countries [1]. The incidence of malignant melanoma, the most dangerous form of skin cancer, is increasing worldwide. The severity of the problem is not only the increased morbidity, but also the ineffectiveness of current treatment options [2].

Platinum-based chemotherapeutic drugs are widely used to treat cancer. Cisplatin (CDDP) is used to treat many types of cancers, including ovarian, testicular, bladder, lung, and head and neck cancer [3]. CDDP is also used to treat melanoma, especially in combination with other drugs such as anvirzel [4,5]. CDDP binds to genomic DNA (gDNA) or mitochondrial DNA (mtDNA) to form platinum-DNA adducts and blocks DNA replication, leading to necrosis or apoptosis [6]. Unfortunately, CDDP therapy is fraught with side effects. The main toxicities resulting from CDDP therapy are neurotoxicity, nephrotoxicity, hepatotoxicity, and ototoxicity [7,8,9,10,11]. 

At present, novel structural analogues of platinum, which can reduce the side effects of CDDP and resistance to chemotherapeutics, as well as improve the effectiveness of anticancer activity, are designed, synthesized, and tested [12,13]. So far, carboplatin and oxaliplatin have been approved and licensed for the treatment of various cancers [14].

Although surgical excision is the fundamental treatment for malignant melanoma of the skin, chemotherapy is used as an adjuvant treatment, especially if melanoma undergoes invasion in the form of metastases. In such a clinical situation, chemotherapy for melanoma remains an oncological challenge. Dacarbazine is considered the gold standard regimen for melanoma. However, in the treatment of melanomas, several active agents, e.g., CDDP, vinblastine, vemurafenib, and temozolomid, are also used [15]. Due to the numerous side effects induced by synthetic chemotherapeutic drugs, natural substances with anticancer properties and their combinations with known chemotherapeutic agents, which produce synergistic and additive effects, are sought.

So far, the antitumor activities of many combinations of CDDP with compounds of natural origin, including thymoquinone [16], baicalein [17], fucoxanthin [18], genistein [19], curcumin [20], quercetin [21], and many others [22], have been investigated. These compounds in combination with CDDP increase the sensitivity of cancer cells to chemotherapy. 

**Figure 1:**
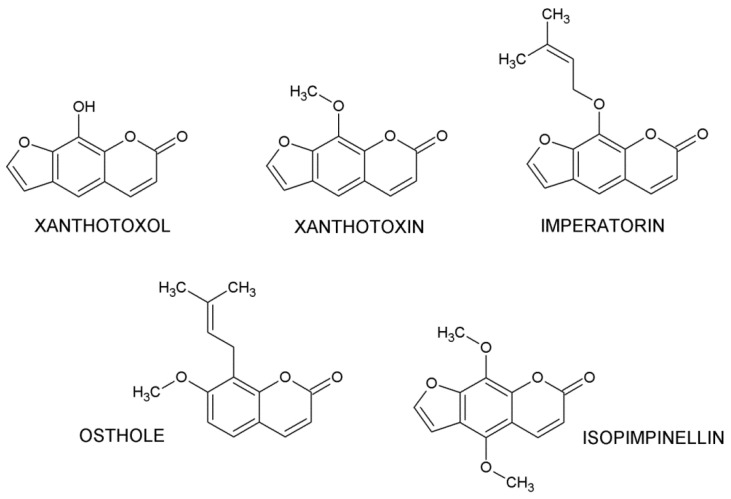
Chemical structure of selected coumarins.

Naturally occurring coumarins are the largest group of benzopyrone derivatives (1,2-benzopyrones or 2*H*-1-benzopyran-2-ones), widespread in plant species belonging to various families, including *Apiaceae, Rutaceae*, *Asteraceae*, *Fabaceae*, *Oleaceae*, *Moraceae*, and *Thymelaeaceae* [23]. These compounds are classified into different groups: simple coumarins such as osthole (OST), furanocoumarins (e.g., xanthotoxin (XIN), xanthotoxol (XOL), isopimpinellin (ISO), and imperatorin (IMP)—Figure 1), pyranocoumarins (e.g., visnadin, xanthyletin, and seselin), dicoumarins, and pyrone-substituted coumarins, such as alternariol, gravelliferone, coumestrol, warfarin, dicumarol, and novobiocin [24]. 

In the last few years, there has been an increased interest in naturally occurring coumarins due to their various biological and pharmacological properties and their low toxicity. It has been shown that they possess anticancer [25], antibacterial [26], antifungal [27], anti-inflammatory and antiviral [28], anticonvulsant [29], antihyperglycemic [30], triglyceride-lowering [31], antioxidant [32], bronchodilator [33], and vasodilator [34] effects. The molecular modeling of coumarins and various types of substitutions in their nuclei, represented by benzo-α-pyrone, may open new directions in the search and design of new, more potent compounds as effective adjuvants for the treatment of various diseases [35]. The interest in coumarins as potential anticancer agents in the treatment of tumors results from both in vitro and in vivo studies reporting that these compounds are effective in preventing the proliferation of bladder cancer [36], colon cancer [37], lung cancer [38], leukemia [39], and breast cancer [40] through different mechanisms of action, including cell cycle arrest, modulation of estrogen receptors, inhibition of DNA-associated enzymes such as topoisomerase [41], inhibition of angiogenesis, several types of heat shock proteins, and activity of enzymes involved in the pathophysiology of cancer, such as telomerase, monocarboxylate transporters, carbonic anhydrase, aromatase, and sulfatase [42], as well as modulation of different signaling pathways, such as the regulation of mitogen-activated protein kinase expression, signal transducers, and activators of transcription 3, phosphatidylinositol-3-kinase/AKT, and nuclear factor kB [43]. It is worth noting that some coumarins and their synthetic derivatives have shown promising activity against several types of cancer in clinical trials [44].

## 2. Results

CDDP and the tested coumarins (OST, XIN, XOL, ISO, and IMP) inhibited the proliferation of human melanoma cells (FM55P and FM55M2) in a concentration-dependent manner when applied separately (Figure S1). Of note, neither phosphate buffered saline (PBS) nor dimethyl sulfoxide (DMSO) (used as solvents in the respective control groups) affected the viability of melanoma cells (data not shown). In the present study, the experimentally derived median inhibitory concentration (IC_50_) values for CDDP, OST, XIN, XOL, ISO, and IMP are presented in Table 1. 

Next, the antiproliferative effects of each of the tested coumarins administered in combination with CDDP to the FM55P and FM55M2 melanoma cell lines were determined. Incubation of the FM55P and FM55M2 cells with different concentrations of both drugs, based on the established IC_50_ values, resulted in a concentration-dependent reduction in cancer cell viability. The test for the parallelism of the concentration–response lines between CDDP and the tested coumarins confirmed that the log-probit lines of these compounds were either nonparallel or parallel to one another (Figure S2).

**Figure 2:**
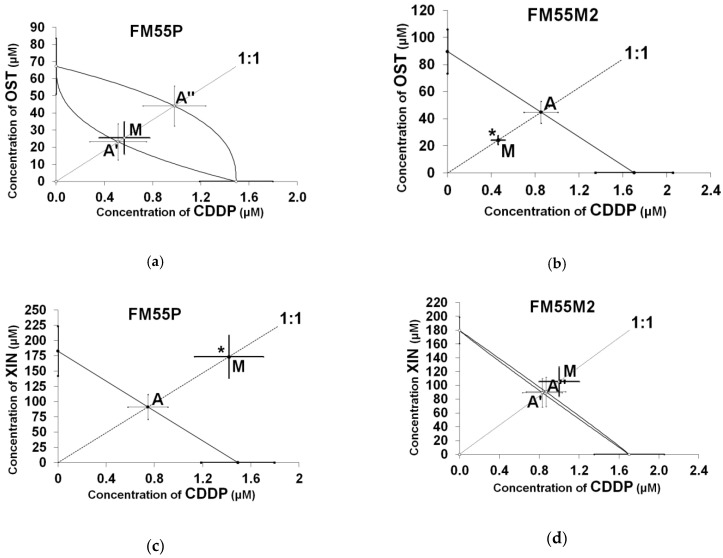

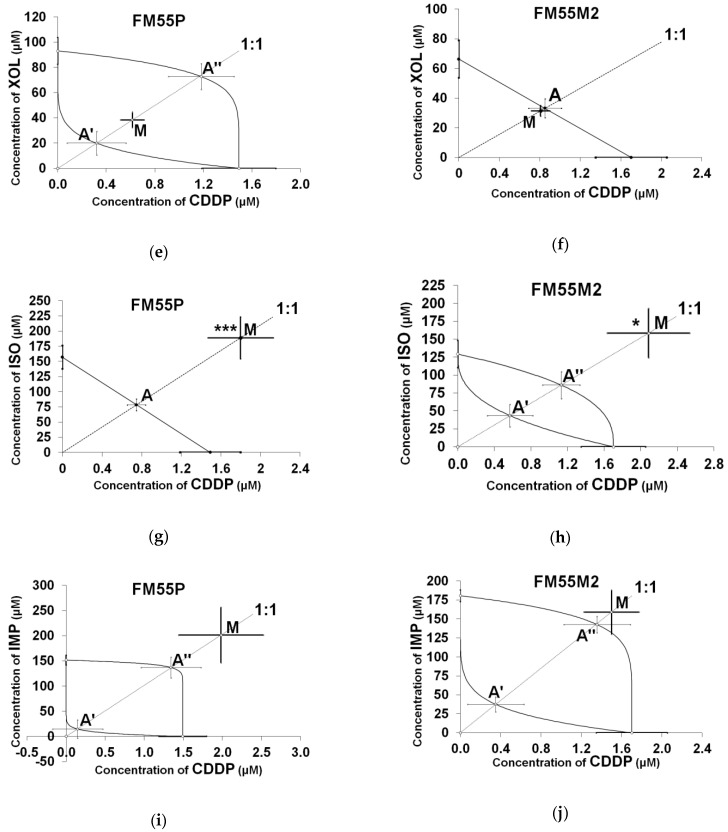
Isobolograms showing additive and synergistic interactions between cisplatin (CDDP) and osthole (OST) for the human melanoma cell line FM55P (**a**) and for cell line FM55M2 (**b**), respectively. Isobolograms showing antagonistic and additive interactions between cisplatin (CDDP) and xanthotoxin (XIN) for the FM55P cell line (**c**) and the FM55M2 cell line (**d**), respectively. Isobolograms illustrating additive interaction between cisplatin (CDDP) and xanthotoxol (XOL) for both FM55P and FM55M2 cell lines (**e**,**f**). Isobolograms showing antagonistic interaction between cisplatin (CDDP) and isopimpinellin (ISO) for both FM55P and FM55M2 cell lines (**g**,**h**). Isobolograms illustrating additive interaction with a tendency toward antagonism between cisplatin (CDDP) and imperatorin (IMP) for both FM55P and FM55M2 cell lines (**i**,**j**). The median inhibitory concentrations (IC_50_) for CDDP and the tested coumarins (OST, XIN, XOL, ISO, and IMP) are plotted on the *x*- and *y*-axes, respectively. The solid lines on the *x-* and *y*-axes represent the S.E. for the IC_50_ values for the studied drugs, when administered alone. The lower and upper isoboles of additivity represent the curves connecting the IC_50_ values of CDDP and the tested coumarins administered alone, if their concentration-response relationships were nonparallel. For collateral concentration-response relationships between CDDP and the studied coumarins, the isobole represents the straight diagonal line connecting the IC_50_ values. The dotted line starting from the point (0,0) corresponds to the fixed ratio of 1:1 for the combination of CDDP with each of the tested coumarins. The points A’ and A” depict the theoretically calculated IC_50 add_ values for both lower and upper isoboles of additivity. The point M represents the experimentally derived IC_50 exp_ value for a total dose of the mixture expressed as proportions of CDDP and each of the tested coumarins that produced a 50% antiproliferative effect in the FM55P and FM55M2 cell lines, as measured in vitro by the MTT assay. * *p* < 0.05 and *** *p* < 0.001 vs. the respective IC_50 add_ values.

Isobolographic analysis of the interactions between CDDP and the tested coumarins (OST, XIN, XOL, ISO, and IMP) revealed that in vitro interactions were synergistic, additive, or antagonistic, depending on the coumarins used. In both cell lines, the FM55P and FM55M2 interactions of CDDP with ISO and XIN were antagonistic, while those of CDDP with XOL and IMP were additive (Table 2 and Table 3, Figure 2). In the FM55P cell line, the interaction of CDDP with OST was additive, but in the FM55M2 cell line, the combination of CDDP with OST was synergistic (Table 2 and Table 3, Figure 2).

## 3. Discussion

All the tested coumarins have antiproliferative activity in various cancer cell lines. For example, OST induces apoptosis and inhibits proliferation in bile duct cancer [45], stomach cancer [46], kidney cancer [47], and lung cancer [48]. The IC_50_ values of OST oscillate from about 75 μM for human ovarian cancer cells [49] to 46.2 µM for lung cancer cells, 42.4 µM for breast cancer cells, 24.8 µM for prostate cancer cells, and 23.2 µM for the human squamous carcinoma cell line A-431 [50]. XIN has antiproliferative activity (10 µg/mL) in the MCF-7 breast cancer cell line [51]. The IC_50_ values of XIN range from above 50 µM for human lung and colon cancer cell lines to 46.8 µM for prostate cancer, 44 µM for the melanoma cell line A375, and 37.8 µM for human squamous carcinoma [50]. Likewise, XOL is cytotoxic for the breast cancer cell line MCF-7, and the IC_50_ for this compound amounted to 11.92 mg/mL [52]. The IC_50_ for XOL is about 25 µM for lung cancer, 37.3 µM for prostate cancer, and above 50 µM for the human squamous carcinoma cell line A431 and melanoma cell line A375 [50]. The IC_50_ values of IMP range from 12.3 µg/mL for central nervous system cancer, 13.7 µg/mL for ovarian cancer, 14.5 µg/mL for melanoma SK-MEL-2 cell line, 16.4 µg/mL for lung cancer, to 19.4 µg/mL for colon cancer [53].

In this study, we evaluated the antiproliferative effects of a simple coumarin (OST) and some selected furanocoumarins (IMP, ISO, XIN, and XOL) to ascertain which of the naturally occurring compounds can be used in the treatment of melanoma. Of note, all the tested coumarins produced antiproliferative effects in both melanoma cell lines, finally resulting in the calculated IC_50_ values. It was observed that the most favorable coumarin was OST, offering 50% anticancer effects with the lowest concentrations, while the highest IC_50_ values were documented for IMP, XIN, and ISO in both melanoma cell lines (Table 1). Considering the IC_50_ values for the studied coumarins, only OST and XOL can be recommended for further characterization of interaction between CDDP and coumarins. CDDP is considered a gold standard in experimental in vitro studies because of referential comparison of the anticancer activity of the compounds tested in this study.

The results of our research indicate that the combination of CDDP and OST is characterized by the most desirable synergistic interaction. The other tested coumarins (belonging to a furanocoumarin group) mostly showed antagonist interactions in combination with CDDP. The exception was XOL, which showed an additive interaction with CDDP and IMP, producing additivity with a slight tendency toward antagonism when combined with CDDP. The results presented here indicate clearly that some of the tested coumarins (OST and XOL) can be used in combination with CDDP because of synergistic and additive interactions. By contrast, ISO, IMP, and XIN should not be recommended as add-on drugs with CDDP. These furanocoumarins produced an antagonistic or additive interaction with a tendency toward antagonism in human melanoma cell lines (FM55P and FM55M2). It should be highlighted that the isobolographic analysis of interaction has been used several times to assess the interactions of various chemical substances with cancer cells [54,55,56,57]. 

In our study, we used two melanoma cell lines to observe any differences in the antiproliferative activity of the tested coumarins. The first melanoma cell line (FM55P) was derived from the primary tumor of the skin, while the second line (FM55M2) was derived from the metastases of melanoma. We confirmed that CDDP when combined with OST exerts a synergistic interaction in terms of anticancer effects on FM55M2. The combination produced a synergistic interaction in the metastatic cell line FM55M2. Although the combination of CDDP and OST exerted an additive interaction with respect to anticancer effects on the primary melanoma cell line FM55P, this combination can be recommended as a treatment option, especially if we are not sure whether melanoma metastases appear. The synergy observed in the human melanoma metastatic cell line (FM55M2) is very beneficial as a treatment option. By contrast, antagonistic interactions in terms of anticancer effects are not recommended because drugs should be used in higher doses/concentrations to eliminate 50% of neoplasmic cells. In such a case, under clinical conditions, patients should receive higher drug doses, and thus, adverse effects may occur more frequently than expected. 

Both interactions, synergy and antagonism, may also be explained in light of their molecular mechanism(s) of the antiproliferative effects influencing the cell cycle. Generally, if two drugs synergistically inhibit proliferation, they probably affect various different phases/sites of the cell cycle, finally contributing to faster apoptosis of the affected cells. In oncology, this effect is highly desired by patients and doctors. By contrast, antagonistic interaction between two anticancer drugs is not favorable, because one of the drugs, in its anticancer activity, probably competes with and reduces the effects produced by the second drug. Another explanation is also possible while considering the fact that one drug can affect different phases of the cell cycle. In such a case, one of the drugs used in the mixture stops/blocks the cell cycle, making the second drug ineffective. If the first drug switches the cell cycle off, the second drug cannot exert its anticancer action, especially if its molecular mechanisms are associated with the transition of cancer cells to another phase of the cell cycle. Thus, one drug can block the activity of the second drug, making the mixture less active than particular drugs when used alone. XIN and ISO are the coumarins that produced antagonistic effects in both melanoma cell lines, primary and metastatic melanoma. XOL and IMP produced additive interaction in both cell lines, primary and metastatic melanoma.

The results presented here (indicating that various coumarins produce various interactions in various cell lines) are a good example illustrating that the screening test can select the most active anticancer agents among the naturally occurring coumarins tested, which can be useful in the treatment of melanoma. This screening among the five naturally occurring coumarins allows us to find the most promising agent, OST. We are fully aware of the fact that translation of the results from this in vitro study to clinical conditions is not so fast as one would expect, but it provides us with the hope that confirmation of this synergistic interaction in other human melanoma cell lines brings us a new option for the treatment of metastatic melanoma in the future, and this study can contribute to bettering our knowledge on melanoma treatment. More advanced studies are required to confirm whether OST can be used as an add-on drug in the treatment of melanoma in in vivo preclinical studies.

The antitumor activity of various naturally occurring compounds belonging to flavonoids, alkaloids, polyphenols, glycosides (including, coumarins), and carotenoids has been demonstrated earlier [16,17,18,19,20,21]. These compounds exhibit different molecular mechanisms affecting various pathways, including NF-κB, Nrf2, Akt, MAPKs, p53, and apoptotic pathways. Additionally, naturally occurring compounds often attenuate CDDP toxicity through their antioxidant and anti-inflammatory effects [22]. Considering the molecular mechanism(s) of action of the most promising combination of CDDP and OST, it should be stressed that the antitumor activity of coumarins is mainly related to the induction of apoptosis through the caspase-dependent mechanism [58]. As regards OST, it was observed that the drug produced upregulation of the ratio of Bax/Bcl-2 proteins and inhibited Akt kinase activity [59]. With respect to CDDP, the drug binds to genomic and mitochondrial DNA, forming platinum–DNA adducts and blocking DNA replication, which consequently leads to necrosis or apoptosis [6]. Thus, it is highly likely that the synergistic interaction between CDDP and OST in terms of their antiproliferative effects in the FM55M2 melanoma cell line resulted from diverse molecular mechanisms of action of the tested drugs. 

It should be stressed that in this study, we did not determine the cytotoxicity of the tested coumarins on normal (healthy) cells. However, some reports revealed that IMP, ISO, XIN, XOL, and OST do not exert cytotoxic effects on normal skin cells [58,59]. Thus, it can be assumed that combination of OST with CDDP can enhance the cytotoxic effect of the latter drug. Numerous experimental studies have indicated that various coumarins increase the sensitivity of cancer cell lines to CDDP therapy.

Another fact should be emphasized while explaining the rationale of combining coumarins with CDDP. It is thought that the resistance of cancer to chemotherapy is one of the major causes of treatment failure and is responsible for over 90% of deaths in cancer patients receiving traditional chemotherapeutics and/or novel target drugs [60]. The overexpression of multi-drug efflux pumps located in the membrane of cancer cells, including P-glycoprotein (P-gp), was found to be one of the principal mechanisms of multidrug resistance (MDR) [61]. The need for combination of chemotherapy with coumarins is highlighted by the fact that coumarins have been shown to play an important role in MDR inversion [62]. For instance, bergapten and XIN synergistically increased the cytotoxicity of CDDP, daunorubicin, or mitoxantrone in the resistant cancer cell lines due to their effects on the MDR downregulation and/or physical inhibition of the ABC efflux pump activity [63]. OST in combination with CDDP markedly inhibited cell proliferation and induced apoptosis in CDDP-resistant cervical cancer cells, such as SiHA/CDDP and CaSki/CDDP compared to CDDP alone treatment [64]. An in vivo study also revealed that OST in combination with CDDP reduced tumor growth and tumor weight in SiHA/CDDP cell-derived xenografts [64]. It has been shown that OST reverses chemoresistance of the studied cancer cells to CDDP through repressing NRF2 expression [64], an oncogenic transcription factor, which has been proven to promote cancer resistance to chemotherapy by regulating downstream MDR-associated protein and drug transporters [65]. Moreover, it has been found that CDDP combined with OST significantly blocks the phosphatidylinositol-3 kinase/AKT signaling pathway, which mediates cell proliferation, cell cycle, and apoptosis [64].

## 4. Materials and Methods 

*Cell culture.* Primary malignant melanoma cells FM55M2 and FM55P were purchased from the European Collection of Authenticated Cell Cultures (ECACC; Salisbury, UK) and cultured in RPMI 1640 medium (Sigma-Aldrich, St. Louis, MO, USA) containing 10% fetal bovine serum (FBS; Sigma-Aldrich, St. Louis, MO, USA) and 1% penicillin/streptomycin (Sigma-Aldrich, St. Louis, MO, USA) in a 37 °C incubator with 5% CO_2_. The cells grew to 80% confluence.

*Cell treatments.* Cell viability was determined using the MTT assay. Cisplatin (CDDP; Sigma-Aldrich, St. Louis, MO, USA) was dissolved in phosphate buffered saline (PBS) with Ca^2+^ and Mg^2+^. The examined coumarins, osthole (OST), xanthotoxin (XIN), xanthotoxol (XOL), isopimpinellin (ISO), and imperatorin (IMP) (all from Sigma-Aldrich, St. Louis, MO, USA), were dissolved in DMSO as stock solutions. The drugs were dissolved to the respective concentrations with culture medium before use. PBS and DMSO had no effect on cell proliferation. Briefly, the FM55M2 and FM55P cells were seeded on a 96-well plate at a density of 10^4^ cells/well and treated with different concentrations of CDDP and five coumarins (OST, XIN, XOL, ISO, and IMP) for 72 h. 

*MTT assay.* Inhibition of cancer cell proliferation was evaluated by an MTT assay. After treatment with the examined drug and naturally occurring coumarins, the cells were incubated at 37 °C for 3 h with 10 μL of MTT solution (5 mg/mL, Sigma-Aldrich, St. Louis, MO, USA). Then, 100 μL of stop solution (10% SDS, 0.01 M HCl) was added to dissolve the crystals in each well. Following a 12 h incubation, the optical densities were determined at 570 nm with a microplate spectrophotometer (Ledetect 96, Labexim, Lengau, Austria). Each treatment was performed in triplicate, and each experiment was repeated 3 times.

*Isobolographic analysis*. Isobolographic analysis is a statistical method allowing the characterization of pharmacodynamic interaction between drugs and chemical substances. This method was performed as described previously [66,67,68]. First, the percentage of inhibition of cell viability with increasing concentrations of CDDP, and 5 naturally occurring coumarins, OST, XIN, XOL, ISO, and IMP (when administrated singly in the melanoma cell lines FM55P and FM55M2), was measured. Then, the concentration–response effects for each investigated anticancer compound (i.e., CDDP, OST, XIN, XOL, ISO, and IMP) were fitted with log-probit linear regression analysis as described by Litchfield and Wilcoxon [69]. The test for the parallelism of concentration–response effect lines for CDDP and each of the naturally occurring coumarins was performed. The types of interactions between CDDP and OST, XIN, XOL, ISO, and IMP in both melanoma cell lines, FM55P and FM55M2, were isobolographically analyzed according to the methodology described elsewhere [66,67,70]. From the experimentally denoted IC_50_ values for the drugs administered alone, median additive inhibitory concentrations for the mixture of CDDP with one of the investigated coumarins (OST, XIN, XOL, ISO, and IMP) at the fixed ratio of 1:1 (IC_50 add_) were calculated, as described earlier [66]. The experimentally derived IC_50 exp_ values for the mixture of CDDP with each of the studied coumarins (at the fixed ratio of 1:1) were determined based on the concentrations of the mixtures of CDDP with one of tested coumarins, inhibiting 50% of cell viability in the melanoma cell lines (FM55P and FM55M2) measured in vitro by the MTT assay. Log-probit analysis was used to determine the experimentally derived IC_50_ and IC_50 exp_ values for CDDP and the tested coumarins (OST, XIN, XOL, ISO, and IMP) when the drugs were administered alone or in combination for the fixed ratio of 1:1 [69]. The difference between the experimentally derived IC_50 exp_ values for the mixture of CDDP with the tested coumarins and the theoretically additive IC_50 add_ values was statistically verified by using the unpaired Student’s *t*-test, as recommended elsewhere [70].

## 5. Conclusions

In conclusion, the synergistic interaction of CDDP with OST observed isobolographically in the human metastatic melanoma cell line (FM55M2) is worthy of recommendation for further intensive investigations to reveal the molecular mechanism(s) of action involved in this interaction. On the other hand, the antagonistic interaction or additive interaction with a tendency toward antagonism between CDDP and ISO, IMP, and XIN should be explained in further in vitro experiments.

## Figures and Tables

**Table 1:** Antiproliferative effect of cisplatin (CDDP) and 5 naturally occurring coumarins (osthole (OST), xanthotoxin (XIN), xanthotoxol (XOL), isopimpinellin (ISO), and imperatorin (IMP)) administrated singly in the FM55P and FM55M2 melanoma cell lines.

Drug	FM55P IC_50_ (µM ± S.E.)	FM55M2 IC_50_ (µM ± S.E.)
CDDP	1.49 ± 0.30	1.70 ± 0.35
OST	67.26 ± 16.35 ^2^	89.58 ± 16.30 ^1^
XIN	182.64 ± 30.59 ^1^	179.74 ± 19.19 ^2^
XOL	93.09 ± 10.68 ^2^	66.27 ± 12.57 ^1^
ISO	156.81 ± 19.08 ^1^	129.36 ± 19.05 ^2^
IMP	151.58 ± 9.85 ^2^	180.53 ± 7.56 ^2^

^1^ Parallel to CDDP; ^2^ nonparallel to CDDP.

**Table 2:** Isobolographic analysis of interactions for parallel concentration-response effects in melanoma cell lines.

Cell Line	Drug Combination	IC_50 exp_ (µM ± S.E.)	*n*_exp_	IC_50 add_ (µM ± S.E.)	*n*_add_	Interaction
FM55P	CDDP + ISO	190.65 ± 34.95 ***	96	79.15 ± 9.69	140	Antagonistic
FM55M2	CDDP + XOL	32.24 ± 3.64	96	33.99 ± 6.46	140	Additive
FM55P	CDDP + XIN	174.95 ± 35.28 *	96	92.07 ± 20.45	140	Antagonistic
FM55M2	CDDP + OST	24.73 ± 3.34 *	96	45.64 ± 8.32	164	Synergistic

* *p* < 0.05 and *** *p* < 0.001 vs. the respective IC_50 add_ values. IC_50 exp_—experimentally-derived IC_50_; *n*_exp_—number of items for experimental mixture that ranged between 4th and 6th probit; IC_50 add_—theoretically additive IC_50_; *n*_add_—number of items calculated for additive mixture.

**Table 3:** Isobolographic analysis of interactions for nonparallel concentration–response effects in melanoma cell lines.

Cell Line	Drug Combination	IC_50 exp_ (µM ± S.E.)	*n*_exp_	L-IC_50 add_ (µM ± S.E.)	*n*_add_	U-IC_50 add_ (µM ± S.E.)	Interaction
FM55P	CDDP + IMP	203.35 ± 55.54	96	14.83 ± 18.64	116	138.36 ± 20.99	Additive
FM55M2	CDDP + IMP	160.55 ± 29.29	96	37.86 ± 10.04	140	143.99 ± 11.10	Additive
FM55M2	CDDP + ISO	160.53 ± 34.71 *	96	43.96 ± 15.76	140	87.10 ± 19.07	Antagonistic
FM55P	CDDP + XOL	39.00 ± 6.20	72	20.29 ± 9.57	116	73.96 ± 10.46	Additive
FM55M2	CDDP + XIN	106.52 ± 21.62	96	90.30 ± 21.05	140	91.57 ± 21.05	Additive
FM55P	CDDP + OST	26.03 ± 9.71	96	23.66 ± 10.85	164	45.09 ± 11.98	Additive

* *p* < 0.05 vs. the respective IC_50 add_ values. L-IC_50 add_, IC_50 add_ for the lower line of additivity; U-IC_50 add_, IC_50 add_ for the upper line of additivity. For more details see the legend to Table 2.

## Data Availability

Data is contained within the article or supplementary materials.

## Supplementary Materials

The following are available online at https://www.mdpi.com/1422-0067/22/2/537/s1.

## References

[1] Jemal A., Bray F., Center M.M., Ferlay J., Ward E., Forman D. (2011). Global cancer statistics. CA Cancer J. Clin..

[2] Sandru A., Voinea S., Panaitescu E., Blidaru A. (2014). Survival rates of patients with metastatic malignant melanoma. J. Med. Life.

[3] Galluzzi L., Vitale I., Michels J., Brenner C., Szabadkai G., Harel-Bellan A., Castedo M., Kroemer G. (2014). Systems biology of cisplatin resistance: Past, present and future. Cell Death Dis..

[4] Apostolou P., Toloudi M., Chatziioannou M., Ioannou E., Knocke D.R., Nester J., Komiotis D., Papasotiriou I. (2013). AnvirzelTM in combination with cisplatin in breast, colon, lung, prostate, melanoma and pancreatic cancer cell lines. BMC Pharmacol. Toxicol..

[5] Fiorentzis M., Kalirai H., Katopodis P., Seitz B., Viestenz A., Coupland S.E. (2018). Electrochemotherapy with bleomycin and cisplatin enhances cytotoxicity in primary and metastatic uveal melanoma cell lines in vitro. Neoplasma.

[6] Achkar I.W., Abdulrahman N., Al-Sulaiti H., Joseph J.M., Uddin S., Mraiche F. (2018). Cisplatin based therapy: The role of the mitogen activated protein kinase signaling pathway. J. Transl. Med..

[7] Astolfi L., Ghiselli S., Guaran V., Chicca M., Simoni E., Olivetto E., Lelli G., Martini A. (2013). Correlation of adverse effects of cisplatin administration in patients affected by solid tumours: A retrospective evaluation. Oncol. Rep..

[8] Dasari S., Tchounwou P.B. (2014). Cisplatin in cancer therapy: Molecular mechanisms of action. Eur. J. Pharmacol..

[9] Manohar S., Leung N. (2018). Cisplatin nephrotoxicity: A review of the literature. J. Nephrol..

[10] Sheth S., Mukherjea D., Rybak L.P., Ramkumar V. (2017). Mechanisms of cisplatin-induced ototoxicity and otoprotection. Front. Cell Neurosci..

[11] Oun R., Moussa Y.E., Wheate N.J. (2018). The side effects of platinum-based chemotherapy drugs: A review for chemists. Dalton Trans..

[12] Riccardi C., Capasso D., Rozza G.M., Platella C., Montesarchio D., Di Gaetano S., Marzo T., Pratesi A., Messori L., Roviello G.N. (2020). Synthesis, DNA binding studies, and antiproliferative activity of novel Pt(II)-complexes with an L-alanyl-based ligand. J. Inorg. Biochem..

[13] Vaquero M., Busto N., Fernández-Pampín N., Espino G., García B. (2020). Appended Aromatic Moieties Determine the Cytotoxicity of Neutral Cyclometalated Platinum(II) Complexes Derived from 2-(2-Pyridyl)benzimidazole. Inorg. Chem..

[14] Ghosh S. (2019). Cisplatin: The first metal based anticancer drug. Bioorg. Chem..

[15] Garbe C., Eigentler T., Keilholz U., Hauschild A., Kirkwood J.M. (2011). Systematic review of medical treatment in melanoma: Current status and future prospects. Oncologist.

[16] Wilson A.J., Saskowski J., Barham W., Yull F., Khabele D. (2015). Thymoquinone enhances cisplatin-response through direct tumor effects in a syngeneic mouse model of ovarian cancer. J. Ovarian Res..

[17] Yu M., Qi B., Xiaoxiang W., Xu J., Liu X. (2017). Baicalein increases cisplatin sensitivity of A549 lung adenocarcinoma cells via PI3K/Akt/NF-kappaB pathway. Biomed. Pharmacother..

[18] Liu C.L., Lim Y.P., Hu M.L. (2013). Fucoxanthin enhances cisplatin-induced cytotoxicity via NFkappaB- mediated pathway and downregulates DNA repair gene expression in human hepatoma HepG2 cells. Mar. Drugs.

[19] Solomon L.A., Ali S., Banerjee S., Munkarah A.R., Morris R.T., Sarkar F.H. (2008). Sensitization of ovarian cancer cells to cisplatin by genistein: The role of NF-kappaB. J. Ovarian Res..

[20] Park B.H., Lim J.E., Jeon H.G., Seo S.I., Lee H.M., Choi H.Y., Jeon S.S., Jeong B.C. (2016). Curcumin potentiates antitumor activity of cisplatin in bladder cancer cell lines via ROS-mediated activation of ERK1/2. Oncotarget.

[21] Zhang X., Guo Q., Chen J., Chen Z. (2015). Quercetin enhances cisplatin sensitivity of human osteosarcoma cells by modulating microRNA-217-KRAS Axis. Mol. Cells.

[22] Sun C.Y., Zhang Q.Y., Zheng G.J., Feng B. (2019). Phytochemicals: Current strategy to sensitize cancer cells to cisplatin. Biomed. Pharmacother..

[23] Majnooni M.B., Fakhri S., Smeriglio A., Trombetta D., Croley C.R., Bhattacharyya P., Sobarzo-Sánchez E., Farzaei M.H., Bishayee A. (2019). Antiangiogenic effects of coumarins against cancer: From chemistry to medicine. Molecules.

[24] Akkol E.K., Genç Y., Karpuz B., Sobarzo-Sánchez E., Capasso R. (2020). Coumarins and coumarin-related compounds in pharmacotherapy of cancer. Cancers.

[25] Venugopala K.N., Rashmi V., Odhav B. (2013). Review on natural coumarin lead compounds for their pharmacological activity. Biomed. Res. Int..

[26] Iqbal P.F., Bhat A.R., Azam A. (2009). Antiamoebic coumarins from the root bark of Adina cordifolia and their new thiosemicarbazone derivatives. Eur. J. Med. Chem..

[27] Tada Y., Shikishima Y., Takaishi Y., Shibata H., Higuti T., Honda G., Ito M., Takeda Y., Kodzhimatov O.K., Ashurmetov O. (2002). Coumarins and gamma-pyrone derivatives from Prangos pabularia antibacterial activity and inhibition of cytokine release. Phytochemistry.

[28] Zhang H.L., Wu X.Y., Mi J., Peng Y.J., Wang Z.G., Liu Y., Wu X.L., Gao Y. (2017). A new anti-inflammatory alkaloid from roots of Heracleum dissectum. Chem. Biodivers..

[29] Luszczki J.J., Andres-Mach M., Glensk M., Skalicka-Wozniak K. (2010). Anticonvulsant effects of four linear furanocoumarins, bergapten, imperatorin, oxypeucedanin, and xanthotoxin, in the mouse maximal electroshock-induced seizure model: A comparative study. Pharmacol. Rep..

[30] Ramesh B., Pugalendi K.V. (2006). Impact of 7-hydroxycoumarin on hepaticmarker enzymes in streptozotocin diabetic rats marker enzymes in streptozocin diabetic rats. Indian J. Pharmacol..

[31] Madhavan G.R., Balraju V., Mallesham B., Chakrabarti R., Lohray V.B. (2003). Novel coumarin derivatives of heterocyclic compounds as lipid-Lowering agents. Bioorg. Med. Chem. Lett..

[32] Torres R., Faini F., Modak B., Urbina F., Labbé C., Guerrero J. (2006). Antioxidant activity of coumarins and flavonols from the resinous exudate of Haplopappus multifolius. Phytochemistry.

[33] Piao X.L., Park I.H., Baek S.H., Kim H.Y., Park M.K., Park J.H. (2004). Antioxidative activity of furanocoumarins isolated from Angelicae dahuricae. J. Ethnopharmacol..

[34] Ramanithrasimbola D., Rakotondramanan D.A., Rasoanaivo P., Randriantsoa A., Ratsimamanga S., Palazzino G., Galeffi C., Nicoletti M. (2005). Bronchodilator activity of Phymatodes scolopendria (Burm) Ching and its bioactive constituent. J. Ethnopharmacol..

[35] Annunziata F., Pinna C., Dallavalle S., Tamborini L., Pinto A. (2020). An Overview of Coumarin as a Versatile and Readily Accessible Scaffold with Broad-Ranging Biological Activities. Int. J. Mol. Sci..

[36] Haghighitalab A., Matin M.M., Bahrami A.R., Iranshahi M., Saeinasab M., Haghighi F. (2014). In vitro investigation of anticancer, cell-cycle-inhibitory, and apoptosis-inducing effects of diversin, a natural prenylated coumarin, on bladder carcinoma cells. Z. Nat. C J. Biosci..

[37] Saidu N.E., Valente S., Bana E., Kirsch G., Bagrel D., Montenarh M. (2012). Coumarin polysulfides inhibit cell growth and induce apoptosis in HCT116 colon cancer cells. Bioorg. Med. Chem..

[38] Kumar M., Singla R., Dandriyal J., Jaitak V. (2018). Coumarin derivatives as anticancer agents for lung cancer therapy: A review. Anticancer Agents Med. Chem..

[39] Lee B.Z., Lee I.S., Pham C.H., Jeong S.K., Lee S., Hong K., Yoo H.M. (2020). Apoptosis in leukemic cells induced by anti-proliferative coumarin isolated from the stem bark of Fraxinus rhynchophylla. J. Microbiol. Biotechnol..

[40] Autore G., Marzocco S., Formisano C., Bruno M., Rosselli S., Jemia M.B., Senatore F. (2015). Cytotoxic activity and composition of petroleum ether extract from Magydaris tomentosa (Desf.) W. D. J. Koch (Apiaceae). Molecules.

[41] Emami S., Dadashpour S. (2015). Current developments of coumarin-based anti-cancer agents in medicinal chemistry. Eur. J. Med. Chem..

[42] Shokoohinia Y., Jafari F., Mohammadi Z., Bazvandi L., Hosseinzadeh L., Chow N., Bhattacharyya P., Farzaei M.H., Farooqi A.A., Nabavi S.M. (2018). Potential Anticancer Properties of Osthol: A Comprehensive Mechanistic Review. Nutrients.

[43] Bruni R., Barreca D., Protti M., Brighenti V., Righetti L., Anceschi L., Mercolini L., Benvenuti S., Gattuso G., Pellati F. (2019). Botanical Sources, Chemistry, Analysis, and Biological Activity of Furanocoumarins of Pharmaceutical Interest. Molecules.

[44] Venkata Sairam K., Gurupadayya B.M., Chandan R.S., Nagesha D.K., Vishwanathan B. (2016). A review on chemical profile of coumarins and their therapeutic role in the treatment of cancer. Curr. Drug Deliv..

[45] Xu X., Liu X., Zhang Y. (2018). Osthole inhibits gastric cancer cell proliferation through regulation of PI3K/AKT. PLoS ONE.

[46] Zhu X., Song X., Xie K., Zhang X., He W., Liu F. (2017). Osthole induces apoptosis and suppresses proliferation via the PI3K/Akt pathway in intrahepatic cholangiocarcinoma. Int. J. Mol. Med..

[47] Liu L., Mao J., Wang Q., Zhang Z., Wu G., Tang Q., Zhao B., Li L., Li Q. (2017). In vitro anticancer activities of osthole against renal cell carcinoma cells. Biomed. Pharmacother..

[48] Xu X.M., Zhang Y., Qu D., Feng X.W., Chen Y., Zhao L. (2012). Osthole suppresses migration and invasion of A549 human lung cancer cells through inhibition of matrix metalloproteinase-2 and matrix metallopeptidase-9 in vitro. Mol. Med. Rep..

[49] Liang J., Zhou J., Xu Y., Huang X., Wang X., Huang W., Li H. (2020). Osthole inhibits ovarian carcinoma cells through LC3-mediated autophagy and GSDME-dependent pyroptosis except for apoptosis. Eur. J. Pharmacol..

[50] Farooq S., Dangroo N.A., Priya D., Banday J.A., Sangwan P.L., Qurishi M.A., Koul S., Saxena A.K. (2014). Isolation, cytotoxicity evaluation and HPLC-quantification of the chemical constituents from Prangos pabularia. PLoS ONE.

[51] Abdel Hafez O.M., Amin K.M., Abdel-Latif N.A., Mohamed T.K., Ahmed E.Y., Maher T. (2009). Synthesis and antitumor activity of some new xanthotoxin derivatives. Eur. J. Med. Chem..

[52] Maneerat W., Prawat U., Saewan N., Laphookhieo S. (2010). New coumarins from Clausena lansium twigs. J. Braz. Chem. Soc..

[53] Kim Y.K., Kim Y.S., Ryu S.Y. (2007). Antiproliferative effect of furanocoumarins from the root of Angelica dahurica on cultured human tumor cell lines. Phytother. Res..

[54] Jarząb A., Luszczki J.J., Guz M., Skalicka-Woźniak K., Hałasa M., Smok-Kalwat J., Polberg K., Stepulak A. (2018). Combination of osthole and cisplatin against rhabdomyosarcoma TE671 cells yielded additive pharmacologic interaction by means of isobolographic analysis. Anticancer Res..

[55] Grabarska A., Luszczki J.J., Nowosadzka E., Gumbarewicz E., Jeleniewicz W., Dmoszyńska-Graniczka M., Kowalczuk K., Kupisz K., Polberg K., Stepulak A. (2017). Histone deacetylase inhibitor SAHA as potential targeted therapy agent for larynx cancer cells. J. Cancer.

[56] Bobiński M., Okła K., Luszczki J.J., Bednarek W., Wawruszak A., Moreno-Bueno G., Dmoszyńska-Graniczka M., Tarkowski R., Kotarski J. (2019). Isobolographic analysis demonstrates the additive and synergistic effects of gemcitabine combined with fucoidan in uterine sarcomas and carcinosarcoma cells. Cancers.

[57] Wawruszak A., Luszczki J.J., Kalafut J., Okla K., Halasa M., Rivero-Muller A., Stepulak A. (2019). Additive Pharmacological Interaction between Cisplatin (CDDP) and Histone Deacetylase Inhibitors (HDIs) in MDA-MB-231 Triple Negative Breast Cancer (TNBC) Cells with Altered Notch1 Activity—An Isobolographic Analysis. Int. J. Mol. Sci..

[58] Grabarska A., Skalicka-Woźniak K., Kiełbus M., Dmoszyńska-Graniczka M., Miziak P., Szumiło J., Nowosadzka E., Kowalczuk K., Khalifa S., Smok-Kalwat J. (2020). Imperatorin as a Promising Chemotherapeutic Agent Against Human Larynx Cancer and Rhabdomyosarcoma Cells. Molecules.

[59] Jarząb A., Grabarska A., Kiełbus M., Jeleniewicz W., Dmoszyńska-Graniczka M., Skalicka-Woźniak K., Sieniawska E., Polberg K., Stepulak A. (2014). Osthole induces apoptosis, suppresses cell-cycle progression and proliferation of cancer cells. Anticancer Res..

[60] Bukowski K., Kciuk M., Kontek R. (2020). Mechanisms of Multidrug Resistance in Cancer Chemotherapy. Int. J. Mol. Sci..

[61] Nanayakkara A.K., Follit C.A., Chen G., Williams N.S., Vogel P.D., Wise J.G. (2018). Targeted inhibitors of P-glycoprotein increase chemotherapeutic-induced mortality of multidrug resistant tumor cells. Sci. Rep..

[62] Ahmed S., Khan H., Aschner M., Mirzae H., Küpeli Akkol E., Capasso R. (2020). Anticancer Potential of Furanocoumarins: Mechanistic and Therapeutic Aspects. Int. J. Mol. Sci..

[63] Mirzaei S.A., Gholamian Dehkordi N., Ghamghami M., Amiri A.H., Dalir Abdolahinia E., Elahian F. (2017). ABC-transporter blockage mediated by xanthotoxin and bergapten is the major pathway for chemosensitization of multidrug-resistant cancer cells. Toxicol. Appl. Pharmacol..

[64] Su J., Zhang F., Li X., Liu Z. (2019). Osthole promotes the suppressive effects of cisplatin on NRF2 expression to prevent drug-resistant cervical cancer progression. Biochem. Biophys. Res. Commun..

[65] Xue D., Zhou X., Qiu J. (2020). Emerging role of NRF2 in ROS-mediated tumor chemoresistance. Biomed. Pharmacother..

[66] Luszczki J.J. (2007). Isobolographic analysis of interaction between drugs with nonparallel dose-response relationship curves: A practical application. Naunyn Schmiedeberg’s Arch. Pharmacol..

[67] Luszczki J.J., Czuczwar S.J. (2006). Biphasic characteristic of interactions between stiripentol and carbamazepine in the mouse maximal electroshock-induced seizure model: A three-dimensional isobolographic analysis. Naunyn Schmiedebergs Arch. Pharmacol..

[68] Luszczki J.J., Filip D., Czuczwar S.J. (2010). Additive interactions of pregabalin with lamotrigine, oxcarbazepine and topiramate in the mouse maximal electroshock-induced seizure model: A type I isobolographic analysis for non-parallel dose-response relationship curves. Epilepsy Res..

[69] Litchfield J.T.J., Wilcoxon F. (1949). A simplified method of evaluating dose-effect experiments. J. Pharmacol. Exp. Ther..

[70] Tallarida R.J. (2001). Drug synergism: Its detection and applications. J. Pharmacol. Exp. Ther..

