# Supplementary figures and images for: Synergy, Additivity, and Antagonism between Cisplatin and Selected Coumarins in Human Melanoma Cells
Source: Int J Mol Sci. 2021 Jan 7;22(2):537. doi: 10.3390/ijms22020537 (PMC7827586; doi:10.3390/ijms22020537)

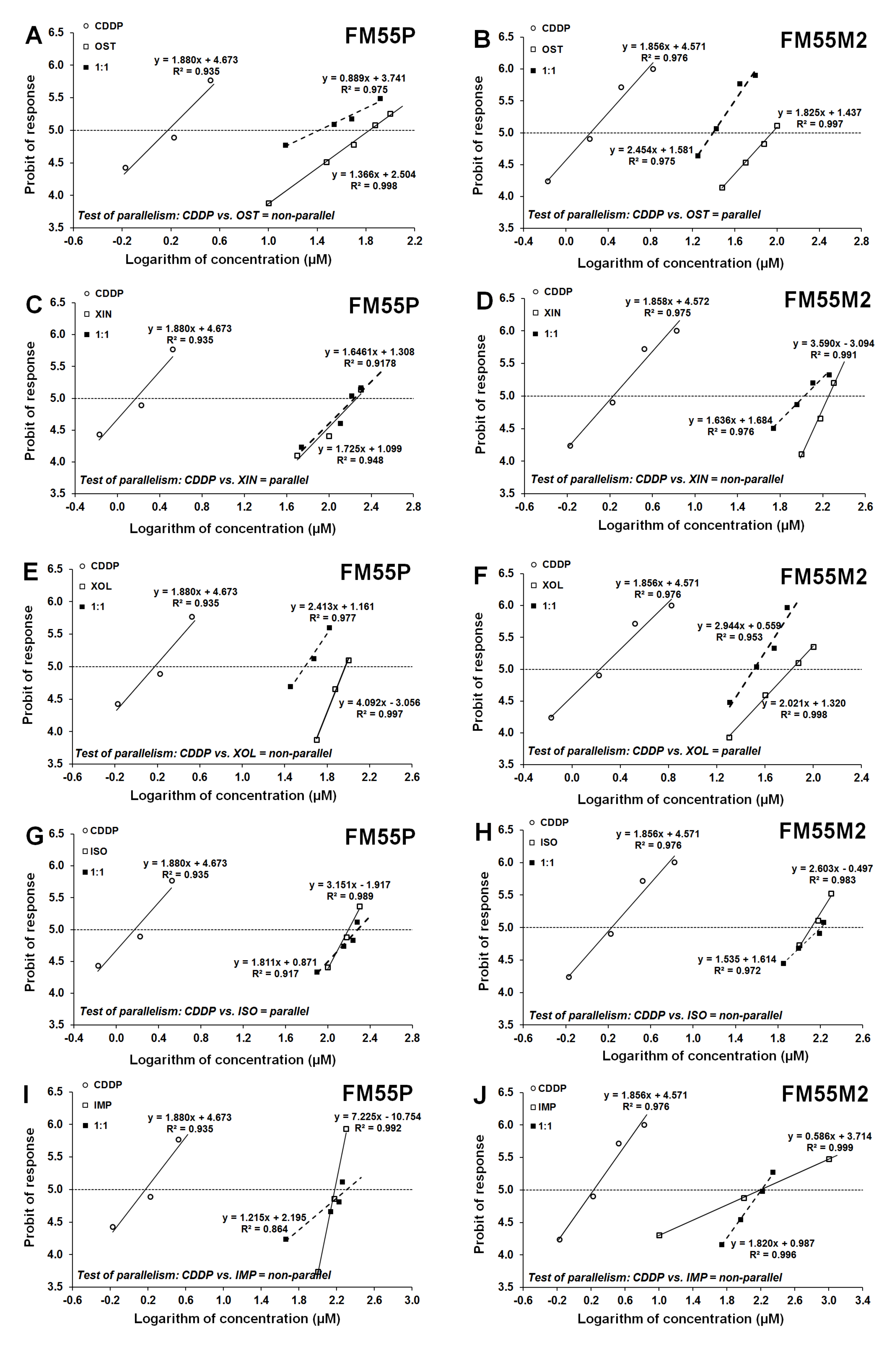

Supplement: Supplementary file 1 [file ijms-22-00537-s001.zip › ijms-10615050supplementary re/Figure S2.tif]
